# Correction: Novel deep reinforcement learning based collision avoidance approach for path planning of robots in unknown environment

**DOI:** 10.1371/journal.pone.0321810

**Published:** 2025-04-01

**Authors:** Raed Alharthi, Iram Noreen, Amna Khan, Turki Aljrees, Zoraiz Ali, Nisreen Innab

There are multiple errors in the published article.

The fifth author’s name is spelled incorrectly. The correct name is: Zoraiz Ali. The correct citation is: Alharthi R, Noreen I, Khan A, Aljrees T, Ali Z, Innab N (2025) Novel deep reinforcement learning based collision avoidance approach for path planning of robots in unknown environment. PLoS ONE 20(1): e0312559. https://doi.org/10.1371/journal.pone.0312559.

The ORCID iD is missing for the second author. Author Iram Noreen’s ORCID iD is 0000-0003-4683-4839 (https://orcid.org/0000-0003-4683-4839).

There is an error in affiliation 2 for author Iram Noreen. The correct affiliation 2 is: Department of Computer Science, Bahria University, Lahore Campus, Islamabad, Pakistan.

The Funding statement for this article is incorrect. The correct Funding statement is as follows: The authors extend their appreciation to the Deanship of Scientific Research, University of Hafr Al Batin, Saudi Arabia, for funding this work through the Research Group Project No. (0025-1443-S).

The following Acknowledgments section is missing from the published article. The Acknowledgments is as follows: The authors would like to express sincere gratitude to AlMaarefa University, Riyadh, Saudi Arabia, for supporting this research.
