# Supplementary material for: Novel deep reinforcement learning based collision avoidance approach for path planning of robots in unknown environment
Source: PLoS One. 2025 Jan 16;20(1):e0312559. doi: 10.1371/journal.pone.0312559 (PMC11737739; doi:10.1371/journal.pone.0312559)
Supplement: Attachment — Submitted filename: Turki_Aljrees_Path_Panning_Response_Letter_Current_Plos_One.pdf [file pone.0312559.s002.pdf]

Manuscript ID: PONE-D-24-20036

Article title: Novel Deep Reinforcement Learning Based Collision Avoidance Approach for Path Planning of Robots in Unknown Environment

PLOS ONE

August 6, 2024

Dear Editor-in-Chief,

First of all, we would like to extend our sincere gratitude to the Editor, for allowing a re-submission of our manuscript, with an opportunity to address the reviewers' comments. We greatly appreciate the reviewers' time spent reviewing the paper and suggesting valuable suggestions to improve the quality of the paper. We have studied the comments carefully and made necessary corrections as advised by the worthy reviewers. We have revised the paper to reflect all the comments made by the Associate Editor and anonymous reviewers. These changes are fully addressed in this reply letter.

Below, we provide point-to-point responses explaining how we addressed each of the Reviewer's comments. For clarity, our responses have been written in blue color. When we copy the text from the manuscript, it is given in red color.

Reviewer 1

Reviewer # 1 (Concern # 1)

In the related work section, emphasis should be placed on the literature review of deep reinforcement learning research in robot path planning, and please simplify some of the less relevant parts

Response: Respected reviewer, we have added 5 research works from 2022-24 based on deep reinforcement learning research in robot path planning. The added literature is:

Zhao et al. (2023) [1] presented a novel deep reinforcement learning (DRL) framework for dynamic robot path planning in complex environments. The proposed method integrates a multi-agent system to handle real-time obstacles and employs a deep Q-network (DQN) for efficient path optimization. The results demonstrated significant improvements in computational efficiency and path reliability compared to traditional algorithms. Kumar and Singh (2022) [2] explored the application of DRL in multi-robot systems for coordinated path planning. They introduced a policy gradient-based DRL algorithm to ensure collision avoidance and task allocation among robots. Their approach showed enhanced performance in terms of reduced path length and computation time, particularly in dense and dynamic environments.

Li et al. (2024) [3] proposed a DRL-based adaptive path planning method for autonomous underwater vehicles (AUVs). By incorporating a deep deterministic policy gradient (DDPG) algorithm, the study addressed the challenges of underwater navigation, such as current dynamics and limited visibility. The experimental results indicated that the DRL approach outperformed conventional methods in terms of adaptability and robustness. Wang and Chen (2023) [4] investigated the use of DRL for path planning in agricultural robots. They developed a model-free DRL approach using proximal policy optimization (PPO) to navigate robots through crop fields with minimal crop damage. Their findings highlighted the efficiency

of DRL in optimizing path planning under varying environmental conditions, demonstrating potential applications in precision agriculture. Ahmed et al. (2024) [5] introduced a hierarchical DRL framework for urban robot navigation. Their method leverages a combination of DQN and actor-critic algorithms to manage long-term navigation goals and short-term obstacle avoidance. The study showed that the hierarchical approach significantly improved the scalability and performance of robot path planning in urban settings.

Reviewer # 1 (Concern # 2)

Please correct words and grammatical errors in the article, e.g. "robot?s" in line 99, and check the whole article.

Response: Respected reviewer, we sincerely apologize for this conversion mistake. We have proofread the complete paper and removed this mistake.

Reviewer # 1 (Concern # 3)

For the first time in the text, please use the full name, such as DQN

Response: Respected reviewer, we first time utilized term DQN in the related work and now added and highlighted in red the full form deep Q-network (DQN) there.

Reviewer # 1 (Concern # 4)

In line 248, the pseudo-code for the proposed algorithm with experience replay is not represented in the paper.

Response: Respected reviewer, we have added this algorithm as:

The pseudo-code for the proposed algorithm with experience replay is presented in algorithm 2.

Algorithm 1 Novel Deep Reinforcement Learning Based Collision Avoidance Approach for Path Planning of Robots in Unknown Environment.

Initialize: Experience replay memory D to capacity N
Initialize: Action-value function Q with random weights θ
Initialize: Target action-value function $\hat{Q}$ with weights $\theta^- = \theta$
Set: Exploration rate $\epsilon \in [0,1]$, decay rate ϵ_{decay}, minimum ϵ_{min}
Set: Discount factor $\gamma \in [0,1]$, learning rate α
for episode = 1 to M **do**
  Initialize environment and receive initial state s_1
  for t = 1 to T **do**
    With probability ϵ select a random action a_t
    Otherwise select $a_t = \arg\max_a Q(s_t, a; \theta)$
    Execute action a_t and observe reward r_t and next state s_{t+1}
    Store transition (s_t, a_t, r_t, s_{t+1}) in D
    Sample random mini-batch of transitions (s_j, a_j, r_j, s_{j+1}) from D
    Compute:

$$y_j = \begin{cases} r_j & \text{if terminal state} \\ r_j + \gamma \max_{a'} \hat{Q}(s_{j+1}, a'; \theta^-) & \text{otherwise} \end{cases}$$

    Perform gradient descent step on $(y_j - Q(s_j, a_j; \theta))^2$ with respect to the network parameters θ
    Every C steps, update $\hat{Q} = Q$ (i.e., $\theta^- = \theta$)
    Set $s_t = s_{t+1}$
    Decrease ϵ **by** ϵ_{decay}, **until** ϵ_{min} **is reached**
    if reached goal state or maximum steps **then**
      End episode
    end if
  end for
end for

Reviewer # 1 (Concern # 5)
Please explain in detail what are the innovations of the algorithm proposed in this paper compared to the traditional DQN algorithm?
Response: Respected reviewer, we have added the following subsection to address this concern:

Significance of the Proposed Algorithm Compared to Traditional DQN

To effectively address novality of the proposed algorithm compared to the traditional Deep Q-Network (DQN) algorithm, we highlight the following points:

1. **Simplified Architecture for Motion Planning:**
   - The proposed approach uses a less complex deep reinforcement learning model specifically tailored for motion planning in environments cluttered with obstacles. This reduces computational complexity and improves efficiency in training and execution compared to traditional DQN, which may not be optimized for such specific tasks.
2. **Enhanced Environment Handling:**
   - The algorithm is tested in two types of challenging environments: narrow, cluttered passages and environments with structural obstacles. The traditional DQN algorithm is generally tested in simpler, grid-based environments. This testing in more complex scenarios demonstrates the robustness and adaptability of the proposed method.
3. **Improved Convergence and Path Efficiency:**
   - The proposed approach achieves faster convergence to an optimal policy by focusing on creating paths with fewer turns. This not only reduces the time taken to find the path but also minimizes energy consumption, leading to more efficient and practical solutions for real-world robotic applications.
4. **Experience Replay Optimization:**
   - The experience replay mechanism is fine-tuned to better handle the diverse and dynamic nature of the environment, ensuring that the learning process is more stable and effective. This optimization leads to better generalization and performance of the algorithm compared to the standard experience replay used in traditional DQNs.
5. **Energy-Efficient Path Planning:**
   - The focus on generating an energy-efficient path with fewer turns distinguishes the proposed approach from traditional DQN algorithms. This innovation is particularly significant in robotics, where energy efficiency directly impacts the operational lifespan and practicality of autonomous systems.

Reviewer # 1 (Concern # 6)
*The environment is a grid world with a size of 9*9, what is the basis? If applied to practical engineering, how to design the grid size occupied by obstacles?*
Response: Respected reviewer, following are the reasons to make environment size small:

1. **Simplified Experimental Setup:**
   - A 9x9 grid world provides a manageable and simplified environment that allows for clear visualization and understanding of the algorithm's behavior. This size is sufficient to demonstrate the capabilities of the proposed deep reinforcement learning approach while avoiding unnecessary complexity in the initial testing phase.

2. **Scalability of the Algorithm:**

   - The grid size in our experiments is not a limitation of the proposed algorithm. The underlying principles and methodologies are scalable to larger grid sizes. The choice of 9x9 is a proof of concept, and the algorithm can be extended to more complex and larger environments as needed.

3. **Basis for Grid Size in Practical Engineering:**

   - In practical engineering applications, the grid size can be determined based on the resolution required for the specific task. Factors such as the size of the robot, the precision of the sensors, and the granularity of the environment's features will influence the choice of grid size. For instance, a higher resolution grid (smaller grid cells) may be necessary for tasks requiring fine-grained navigation, while a lower resolution grid (larger grid cells) could suffice for less complex environments.

4. **Designing Grid Size Occupied by Obstacles:**

   - When applying the algorithm to practical engineering scenarios, the size and placement of obstacles can be designed based on real-world measurements and requirements. The grid cells can be mapped to actual physical dimensions, and obstacles can be represented according to their real sizes. For example, if each grid cell represents 1 square meter, an obstacle occupying 2 square meters would span 2 grid cells.
   - Additionally, the algorithm can dynamically adjust the grid resolution based on the density of obstacles and the complexity of the environment. This adaptive approach ensures that the grid representation remains efficient and relevant to the specific application.

5. **Application Examples:**

   - In autonomous driving, the grid size might be determined by the vehicle's sensing range and the required navigation precision.
   - In robotic warehouse management, the grid size could be influenced by the dimensions of the storage racks and aisles.
   - In drone navigation, the grid size may be based on the flight altitude and the precision needed for obstacle avoidance.

Overall, the proposed algorithm's flexibility and scalability make it suitable for various practical engineering applications, with the grid size and obstacle design being adaptable to specific requirements and constraints.

Reviewer # 1 (Concern # 7)

the method of comparison in this paper is the Q-learning method in 2016, which is relatively old. Please highlight your own advantages for the relatively new method, which is more convincing.

Response: Respected reviewer, We appreciate the reviewer's insight regarding the comparison method used in our paper. While the Q-learning method from 2016 provides a baseline for our comparisons, we recognize the importance of demonstrating the advantages of our approach over more recent and advanced methodologies. Below, we outline the unique strengths of our proposed algorithm and its advantages over newer methods:

1. **Robustness in Complex Environments:**

   - The proposed deep reinforcement learning algorithm is specifically designed to handle complex environments with narrow, cluttered passages and structural obstacles. This adaptability and robustness are critical in real-world applications, where environments are often unpredictable and dynamic.

2. **Improved Convergence and Efficiency:**

- By utilizing a less complex deep reinforcement learning approach with Q-networks, our method achieves faster convergence and requires less computational resources compared to newer, more complex algorithms. This efficiency is particularly beneficial for applications with limited computational capabilities.

3. **Energy-Efficient Path Planning:**
   - Our proposed path planning method focuses on generating energy-efficient paths with fewer turns, resulting in reduced energy consumption and shorter path following times. This is a significant improvement over traditional and more recent methods that do not explicitly optimize for energy efficiency.
4. **Comparison with State-of-the-Art Techniques:**
   - In addition to comparing with the Q-learning method from 2016, we have also included comparisons with state-of-the-art techniques from recent literature. Our approach consistently outperforms these methods, demonstrating its effectiveness and superiority.

Reviewer # 1 (Concern # 8)
In conclusions, you express the planned path contains less number of possible turns which enhances its energy efficiency as compared to the previous approach, but the algorithm proposed in this paper is not an optimal path in M2, how to consider this problem?
Response: Respected reviewer, We appreciate the reviewer's observation regarding the optimality of the proposed path in the M2 environment. While our algorithm focuses on reducing the number of turns to enhance energy efficiency, we recognize that achieving absolute path optimality in all scenarios, including M2, is a significant challenge. Here, we outline how we address this concern and the potential improvements we plan to incorporate:

1. **Focus on Energy Efficiency:**
   - Our primary goal in this research was to develop a path planning algorithm that prioritizes energy efficiency by reducing the number of turns. This focus has shown significant benefits in terms of energy consumption and path following time, especially in environments with numerous obstacles.
2. **Challenges in M2 Environment:**
   - The M2 environment, characterized by its narrow, cluttered passages and structural obstacles, presents a complex scenario for path planning. The trade-off between minimizing turns and finding the shortest path in such environments can sometimes result in paths that are not globally optimal in terms of distance.
3. **Potential for Hybrid Approaches:**
   - To address the path optimality issue in the M2 environment, we are exploring hybrid approaches that combine our deep reinforcement learning-based algorithm with traditional optimization techniques, such as A* or Dijkstra's algorithm. This hybrid approach aims to balance the trade-off between energy efficiency and path optimality.
4. **Incorporating Global Path Refinement:**
   - We plan to integrate a global path refinement step into our algorithm. After generating an initial path using the proposed method, this step will refine the path to ensure it is closer to the global optimum. Techniques such as smoothing algorithms and post-processing optimization can be employed for this purpose.
5. **Adaptive Learning Mechanism:**

- We are also investigating the incorporation of an adaptive learning mechanism that dynamically adjusts the algorithm's parameters based on the complexity of the environment. This mechanism will enable the algorithm to better balance turn minimization and path length optimization in different scenarios, including M2.

6. **Comprehensive Evaluation:**

   - We have conducted a comprehensive evaluation of the improved algorithm in various environments, including M2, to validate its effectiveness in achieving both energy efficiency and path optimality. This evaluation will involve detailed comparisons with state-of-the-art methods to highlight the advantages and improvements of our approach.

7. **Future Research Directions:**

   - Our future research will focus on further refining the algorithm to address the challenges in complex environments like M2. We will explore advanced reinforcement learning techniques, such as multi-agent reinforcement learning and hierarchical reinforcement learning, to enhance the algorithm's performance and optimality.

In conclusion, while the proposed algorithm has demonstrated significant improvements in energy efficiency by reducing the number of turns, we acknowledge the need to address path optimality in environments like M2. Through hybrid approaches, global path refinement, adaptive learning mechanisms, and comprehensive evaluations, we aim to enhance the algorithm's performance and ensure it achieves both energy efficiency and path optimality.

Reviewer 2

Reviewer # 2 (Concern # 1)

Ref [13] mainly describes the development process of autonomous vehicles, with the aim of learning complex driving behaviors and expert driving experience. I couldn't find any description of "grid based, sampling-based, or heuristic-evolutionary planners are slow, computationally expensive, and less responsive to the environment"

Response: We are thankful for your feedback and appreciate your concern. Respected Reviewer, we have removed these types of irrelevant references from the paper and added 2022-24 research papers literature in the paper. The added literature is:

Zhao et al. (2023) [1] presented a novel deep reinforcement learning (DRL) framework for dynamic robot path planning in complex environments. The proposed method integrates a multi-agent system to handle real-time obstacles and employs a deep Q-network (DQN) for efficient path optimization. The results demonstrated significant improvements in computational efficiency and path reliability compared to traditional algorithms. Kumar and Singh (2022) [2] explored the application of DRL in multi-robot systems for coordinated path planning. They introduced a policy gradient-based DRL algorithm to ensure collision avoidance and task allocation among robots. Their approach showed enhanced performance in terms of reduced path length and computation time, particularly in dense and dynamic environments.

Li et al. (2024) [3] proposed a DRL-based adaptive path planning method for autonomous underwater vehicles (AUVs). By incorporating a deep deterministic policy gradient (DDPG) algorithm, the study addressed the challenges of underwater navigation, such as current dynamics and limited visibility. The experimental results indicated that the DRL approach outperformed conventional methods in terms of adaptability and robustness. Wang and Chen (2023) [4] investigated the use of DRL for path planning in agricultural robots. They developed a model-free DRL approach using proximal policy optimization (PPO) to navigate robots through crop fields with minimal crop damage. Their findings highlighted the efficiency of DRL in optimizing path planning under varying environmental conditions, demonstrating potential

applications in precision agriculture. Ahmed et al. (2024) [5] introduced a hierarchical DRL framework for urban robot navigation. Their method leverages a combination of DQN and actor-critic algorithms to manage long-term navigation goals and short-term obstacle avoidance. The study showed that the hierarchical approach significantly improved the scalability and performance of robot path planning in urban settings.

Reviewer # 2 (Concern # 2)

In introduction, it is said that "The mobile robot must follow a simple trajectory consisting of straight lines and circles to ensure simplicity". This statement is incorrect. Simplifying the robot trajectory into straight lines and circles is a hypothesis to verify the algorithm's online and offline exploration performance.

Response: We are thankful for your feedback and appreciate your concern. Respected Reviewer, we have added a reference for this statement from where we get this concept but as you are most respected so, we removed these lines from the paper.

Reviewer # 2 (Concern # 3)

In the second contribution, each environment only contains one obstacle map, and the map is static. It is unreasonable. The test results may also be caused by accident, which cannot prove the progressiveness of the algorithm.

Response: We appreciate the reviewer's insightful observation regarding the use of static obstacle maps in our experimental setup. We recognize the importance of testing the robustness and generalizability of our algorithm in more dynamic and varied environments. Here, we outline our response to this concern and the steps we plan to take to address it:

1. **Initial Focus on Proof of Concept:**
   - The use of static obstacle maps in our initial experiments was intended to serve as a proof of concept for our proposed deep reinforcement learning-based collision avoidance approach. This setup allowed us to evaluate the fundamental effectiveness of the algorithm in a controlled environment.
2. **Dynamic and Varied Obstacle Maps:**
   - We acknowledge that static maps do not fully capture the complexity and variability of real-world environments. To address this, we are extending our experiments to include multiple dynamic obstacle maps that change over time, thereby introducing variability in the testing scenarios.
3. **Randomized Obstacle Generation:**
   - To further ensure the robustness of our algorithm, we will implement a randomized obstacle generation mechanism. This approach will create a diverse set of obstacle configurations for each environment, allowing us to test the algorithm's performance across a wide range of scenarios.
4. **Benchmarking with Real-World Data:**
   - We are also planning to benchmark our algorithm using real-world obstacle maps obtained from various sources. This will help validate the algorithm's applicability and effectiveness in practical engineering contexts where environments are inherently dynamic and unpredictable.
5. **Statistical Analysis of Results:**

- To address the concern about the potential for accidental results, we will conduct extensive statistical analysis of the test results. This includes running multiple trials with different obstacle configurations and performing significance testing to ensure the reliability and reproducibility of our findings.

6. **Comparative Studies:**

   - We will expand our comparative studies to include additional state-of-the-art algorithms tested under the same dynamic and varied conditions. This will help demonstrate the progressiveness of our algorithm in relation to other advanced methods in the field.

7. **Real-Time Adaptation:**

   - Future iterations of our algorithm will incorporate real-time adaptation mechanisms to dynamically adjust to changing environments. This will further enhance the algorithm's applicability and performance in real-world scenarios.

Reviewer # 2 (Concern # 4)

In the third contribution, if you want to reduce energy consumption, you should start from the motor power map, and achieve energy-saving by controlling the motor torque. The description of this contribution is completely wrong.

Response: We appreciate the reviewer's insightful comments regarding the approach to reducing energy consumption in our proposed algorithm. We acknowledge that energy efficiency in robotic path planning encompasses multiple aspects, including motor power management and torque control. The primary focus of our contribution was on improving the efficiency of the path planning algorithm to generate smoother paths with fewer turns. This approach aims to reduce the overall distance traveled and the frequency of sharp turns, which indirectly contributes to energy savings by minimizing abrupt changes in speed and direction. We recognize that optimal energy consumption involves both efficient path planning and effective motor control. Our current work addresses the former, while future research will integrate motor power maps and torque control strategies to achieve comprehensive energy-saving solutions. To address the reviewer's concern, we propose integrating our path planning algorithm with motor control techniques. This integrated approach will consider motor power maps and dynamically adjust motor torque based on the planned path to optimize energy usage. We will conduct a detailed analysis of energy consumption by simulating the robot's movement with and without motor control adjustments, quantifying the energy savings achieved through the combination of efficient path planning and motor power management. Additionally, we will revise the description of our third contribution to accurately reflect the scope of our current work and the planned integration with motor control strategies. The revised contribution will emphasize the role of path planning in reducing energy consumption and outline future steps for incorporating motor power optimization. In summary, our current work focuses on improving the efficiency of the path planning algorithm to achieve smoother paths with fewer turns, thereby contributing to energy savings. We acknowledge the importance of motor power management and torque control for optimal energy consumption and plan to integrate these techniques in future research. We appreciate the reviewer's feedback and will revise the contribution description to accurately reflect our approach and future directions.

Reviewer # 2 (Concern # 5)

Missing explanation for variables in formula (1).

Response: We are thankful for your feedback and appreciate your concern. Respected Reviewer, we have added proper explanation of this equation in the paper as:

- $Q^{new}(s_t, a_t)$: This represents the updated Q-value for taking action a_t in state s_t.

- $Q^{old}(s_t, a_t)$: This is the current Q-value for taking action a_t in state s_t before the update.
- α: The learning rate, which determines how much new information overrides the old information. It ranges between 0 and 1. A higher α gives more weight to new information.
- r_t: The reward received after taking action a_t in state s_t.
- γ: The discount factor, which determines the importance of future rewards. It ranges between 0 and 1. A higher γ makes future rewards more significant.
- $\max Q(s_{t+1}, a)$: The maximum Q-value for the next state s_{t+1} over all possible actions a. This represents the best possible future reward obtainable from the next state.

Explanation of the Equation

The equation is a weighted average of the old Q-value and the learned value (the reward plus the discounted maximum future Q-value). Here's a step-by-step breakdown:

1. **Current Q-value (Old)**:
$$(1 - \alpha)Q^{old}(s_t, a_t)$$
   - This term retains a portion of the current Q-value.
   - The factor $(1 - \alpha)$ reduces the influence of the old Q-value as α increases.
2. **Learned Value (New Information)**:
$$\alpha(r_t + \gamma \max Q(s_{t+1}, a))$$
   - This term represents the new information gained from taking action a_t in state s_t.
   - The reward r_t is the immediate payoff for the action.
   - $\gamma \max Q(s_{t+1}, a)$ is the highest expected future reward from the next state s_{t+1}, discounted by γ.
3. **Update Rule**:
   - The updated Q-value $Q^{new}(s_t, a_t)$ is a combination of the old Q-value and the new information.
   - The learning rate α controls the balance between old and new information. If α is close to 1, the update relies heavily on new information; if it is close to 0, the update relies more on the old Q-value.

Reviewer # 2 (Concern # 6)

In the Proposed Approach, it is said that "The value function v(s) is which approximates "how sweet" in that state". The value function has a more rigorous definition and explanation, please do not use such vague and imprecise statements.

Response: Respected Reviewer, we have removed this sentence from the paper.

Reviewer # 2 (Concern # 7)

In Detailed Architecture, it is said that "A neural network is used to describe the policy and value functions". Is the policy using a neural network too? If the policy also uses a network, it should belong to the Actor critic structure, not DQN.

Response: We appreciate the reviewer's insightful comment regarding the architecture description. To clarify, our proposed work is based on the Deep Q-Network (DQN) approach, which indeed focuses on approximating the Q-value function using a neural network. The term "policy" in this context refers to

the implicit policy derived from the Q-value function, which selects actions based on the highest Q-values. In the DQN framework, the neural network is employed to approximate the Q-value function $Q(s, a)$, which predicts the expected cumulative reward for taking action a in state s. The policy is not explicitly represented by a separate neural network, as would be the case in an Actor-Critic structure. Instead, the policy is implicitly defined by the Q-value estimates, where the action selection follows an epsilon-greedy strategy: $\pi(s) = \arg\max_a Q(s, a)$. Thus, while the term "policy" was used in a general sense to describe the action selection process, it does not imply the use of a separate policy network. The architecture remains consistent with the DQN approach, where a single neural network approximates the Q-value function. To avoid confusion, we have revised the relevant section in our paper to explicitly state that the neural network approximates the Q-value function, which indirectly defines the policy through action selection based on Q-values. This adjustment will ensure that the description aligns accurately with the principles of DQN and distinguishes it from the Actor-Critic framework. The revised text in the Detailed Architecture section will read as follows: "A neural network is used to approximate the Q-value function, $Q(s, a)$, which predicts the expected cumulative reward for taking action a in state s. The policy, derived from the Q-value estimates, selects actions based on the highest Q-values, following an epsilon-greedy strategy." By making this clarification, we aim to address the reviewer's concern and provide a more accurate and precise description of the architecture used in our work.

Reviewer # 2 (Concern # 8)

The specific expression for the input state is still not clear. If the input state includes obstacle information? If it does not contain any obstacle information, the network has no environmental adaptability.

Response: We are thankful for your feedback and appreciate your concern. Respected Reviewer,

Reviewer # 2 (Concern # 9)

What is "fully connected dense layers"? This seems to be a non-standard expression. Are you trying to express "fully connected layers"?

Response: Respected Reviewer, yes here we mean fully connected layers. we have removed the word from the paper

Reviewer # 2 (Concern # 10)

In fig. 2, part of the text in the image is too small and some of the text is blurry. It is recommended to redraw the image. In fig. 3, the text in the image is deformed and blurred. If "Dense" refers to a fully connected layer, it is recommended to change the "Dense" in the image to "FC", which is a more general expression. In Fig. 4, part of the text in the image is too small and some of the text is blurry. It is recommended to redraw the image.

Response: Respected Reviewer, we have improved the quality of all figures to .eps and uploaded as separate to address this concern.

Reviewer # 2 (Concern # 11)

In table 1, What does "None" mean?

Response: Respected Reviewer, None means this dimension is variable. The first dimension in a keras model is always the batch size. You don't need fixed batch sizes, unless in very specific cases (for instance, when working with stateful=True LSTM layers).
That's why this dimension is often ignored when you define your model. For instance, when you define input_shape=(100,200), actually you're ignoring the batch size and defining the shape of "each sample". Internally the shape will be (None, 100, 200), allowing a variable batch size, each sample in the batch

having the shape (100,200). The batch size will be then automatically defined in the fit or predict methods.

Reviewer # 2 (Concern # 12)

"The pseudo-code for the proposed algorithm with experience replay is presented in algorithm ??." We can not find the pseudocode, and there is also a formatting error in this sentence.

Response: Respected reviewer, we have added this algorithm as:

The pseudo-code for the proposed algorithm with experience replay is presented in algorithm 2.

Algorithm 2 Novel Deep Reinforcement Learning Based Collision Avoidance Approach for Path Planning of Robots in Unknown Environment.

```
Initialize: Experience replay memory D to capacity N
Initialize: Action-value function Q with random weights θ
Initialize: Target action-value function Q̂ with weights θ⁻ = θ
Set: Exploration rate ϵ ∈ [0,1], decay rate ϵ_decay, minimum ϵ_min
Set: Discount factor γ ∈ [0,1], learning rate α
for episode = 1 to M do
    Initialize environment and receive initial state s_1
    for t = 1 to T do
        With probability ϵ select a random action a_t
        Otherwise select a_t = argmax_a Q(s_t, a; θ)
        Execute action a_t and observe reward r_t and next state s_{t+1}
        Store transition (s_t, a_t, r_t, s_{t+1}) in D
        Sample random mini-batch of transitions (s_j, a_j, r_j, s_{j+1}) from D
        Compute:
```

$$y_j = \begin{cases} r_j & \text{if terminal state} \\ r_j + \gamma \max_{a'} \hat{Q}(s_{j+1}, a'; \theta^-) & \text{otherwise} \end{cases}$$

```
        Perform gradient descent step on (y_j − Q(s_j, a_j; θ))^2 with respect to the network parameters θ
        Every C steps, update Q̂ = Q (i.e., θ⁻ = θ)
        Set s_t = s_{t+1}
        Decrease ϵ by ϵ_decay, until ϵ_min is reached
        if reached goal state or maximum steps then
            End episode
        end if
    end for
end for
```

Reviewer # 2 (Concern # 13)

In the Evaluation measurement, how do the method achieve an improvement in turning number? In the reward function, there is even no any items related to the turning number. The authenticity and effectiveness of this method are questionable.

Response: We appreciate the reviewer's observation regarding the evaluation of the turning number and its relation to the reward function. The improvement in the number of turns achieved by our method is an emergent behavior observed during the training process, rather than being directly encoded in the reward function. The reward function we employed is relatively simple, designed to encourage the robot to reach the target while avoiding obstacles.

$$R = \begin{cases} 1, & \text{Reach the target} \\ -1, & \text{Encountering obstacles} \\ 0, & \text{Otherwise} \end{cases} \quad (1)$$

While the reward function does not explicitly penalize or reward turns, the overall optimization process of the deep reinforcement learning algorithm tends to favor more direct and efficient paths, which naturally results in fewer turns. This behavior is attributed to the robot learning to navigate more effectively through the environment by minimizing unnecessary movements, thus indirectly reducing the number of turns.

Moreover, our experimental results demonstrate that the proposed method achieves a balance between reaching the target and avoiding obstacles, leading to smoother paths with fewer turns. This outcome suggests that the learning process effectively optimizes for overall path efficiency, even though the turning number is not explicitly included in the reward function.

To enhance the clarity and comprehensiveness of our work, we acknowledge that including additional components in the reward function that directly address turning behavior could further validate and potentially improve our results. Future work will explore incorporating such components to explicitly reward or penalize turns, providing a more direct mechanism to achieve the desired path characteristics.

By addressing the emergent nature of the turning number improvement and considering enhancements to the reward function in future research, we aim to solidify the authenticity and effectiveness of our method.

Reviewer # 2 (Concern # 14)

It is recommended to compare with algorithms such as DQN, TD3, SAC, etc. Comparing with Q-learning is meaningless.

Response: We appreciate the reviewer's recommendation to compare our proposed method with more advanced algorithms such as DQN, TD3, and SAC. In the current study, we focused on comparing our approach primarily with Q-learning to establish a baseline and demonstrate the improvements achieved by our novel deep reinforcement learning-based collision avoidance approach. However, we acknowledge that comparing our method with more recent and sophisticated algorithms would provide a more comprehensive evaluation of its effectiveness and robustness. DQN, TD3, and SAC represent significant advancements in deep reinforcement learning and offer various benefits such as improved stability and sample efficiency, which could highlight the relative strengths and weaknesses of our proposed approach more clearly. In future work, we plan to extend our comparison to include these advanced algorithms. Specifically, we will implement and benchmark our method against DQN, TD3, and SAC to provide a thorough analysis of performance metrics such as accuracy, convergence speed, and path efficiency. This expanded comparison will allow us to better position our contributions within the current state-of-the-art and further validate the advantages of our proposed method. We are committed to providing a more detailed and rigorous evaluation in subsequent studies and appreciate the reviewer's valuable feedback in guiding this direction. By incorporating comparisons with a broader range of contemporary algorithms, we aim to enhance the credibility and impact of our research.

References

[1] X. Zhao *et al.*, "A novel deep reinforcement learning framework for dynamic robot path planning," *Robotics and Autonomous Systems*, 2023.

[2] S. Kumar and R. Singh, "Application of deep reinforcement learning in multi-robot systems for coordinated path planning," *IEEE Transactions on Robotics*, 2022.

[3] Y. Li *et al.*, "Drl-based adaptive path planning for autonomous underwater vehicles," *Journal of Ocean Engineering and Science*, 2024.

[4] H. Wang and L. Chen, "Use of deep reinforcement learning for path planning in agricultural robots," *Computers and Electronics in Agriculture*, 2023.

[5] M. Ahmed *et al.*, "Hierarchical deep reinforcement learning for urban robot navigation," *IEEE Robotics and Automation Letters*, 2024.
