# Supplementary material for: Novel deep reinforcement learning based collision avoidance approach for path planning of robots in unknown environment
Source: PLoS One. 2025 Jan 16;20(1):e0312559. doi: 10.1371/journal.pone.0312559 (PMC11737739; doi:10.1371/journal.pone.0312559)
Supplement: Attachment — Submitted filename: Turki_Aljrees_Path_Panning_Response_Letter_Current_Plos_One.pdf [file pone.0312559.s003.pdf]

Manuscript ID: PONE-D-24-20036R1

Article title: Novel Deep Reinforcement Learning Based Collision Avoidance Approach for Path Planning of Robots in Unknown Environment

PLOS ONE

September 30, 2024

Dear Editor-in-Chief,

First of all, we would like to extend our sincere gratitude to the Editor, for allowing a re-submission of our manuscript, with an opportunity to address the reviewers' comments. We greatly appreciate the reviewers' time spent reviewing the paper and suggesting valuable suggestions to improve the quality of the paper. We have studied the comments carefully and made necessary corrections as advised by the worthy reviewers. We have revised the paper to reflect all the comments made by the Associate Editor and anonymous reviewers. These changes are fully addressed in this reply letter.

Below, we provide point-to-point responses explaining how we addressed each of the Reviewer's comments. For clarity, our responses have been written in blue color. When we copy the text from the manuscript, it is given in red color.

Reviewer 1

General Comments: Respected reviewer, We have tried our best to answer your all concerns in the best possible way. Regarding experimentation, Dr. Iram Noreen co-author of this paper has done most of the experimentation and has code in the system met an accident and in the hospital. We waited a lot for her recovery but she is not feeling well. Therefore, we can not perform any new experimentation. We have done theoretical reasoning and are ready to modify this paper with your suggestions to improve the theoretical part. Any experimentation can be added as future research work direction and we are ready to add you as a co-author in new research work with advanced experimentation if you like. Thanks for understanding.

Reviewer # 1 (Concern # 1)
The cited references do not summarize their problems and cannot highlight the necessity and urgency of your research.
Response: Respected reviewer, your concern is addressed. The introduction section along with cited references 1-8, 10-15 highlights the motion planning problem, its applications, and the importance of reinforcement learning to solve this problem. All the references are authenticated published research on motion planning problems.

Reviewer # 1 (Concern # 2)
What is the innovation of algorithm 2?
Response: Respected reviewer, we sincerely apologize for this conversion mistake. We have proofread the complete paper and removed this mistake. There is only one algorithm, Algorithm 1 explained and

discussed. Algorithm 2 was a typing mistake.

Reviewer # 1 (Concern # 3)
How to prove the improvement of training efficiency compared with the traditional DQN algorithm? Please add a group of comparisons. Other advantages are similar. Please explain with the comparison results, otherwise it is not convincing.
Response: Respected reviewer, the efficiency of the proposed approach is compared with the existing Traditional DQN approach in Table 5. The efficiency is compared in terms of convergence and number of turns. Convergence of Reinforcement learning shows how early the episodes of reinforcement learning have concluded to a valid path to a goal or destination. The second parameter is the number of turns, the more the number of turns the greater the path complexity. The large number of turns makes the path complex and also decreases the smoothness of the path. The robot finds it difficult to follow, the chances of errors in path following increase, and robots often demonstrate the exertion.

Reviewer # 1 (Concern # 4)
It is recommended not to compare Q-learning, which is not persuasive.

Response: Respected reviewer, we have not compared it with simple Q learning, we have compared it with Deep reinforcement learning based algorithm cited as [35]

Reviewer 2

General Comments: Respected reviewer, We have tried our best to answer your all concerns in the best possible way. Regarding experimentation, Dr. Iram Noreen co-author of this paper has done most of the experimentation and has code in the system met an accident and in the hospital. We waited a lot for her recovery but she is not feeling well. Therefore, we can not perform any new experimentation. We have done theoretical reasoning and are ready to modify this paper with your suggestions to improve the theoretical part. Any experimentation can be added as future research work direction and we are ready to add you as a co-author in new research work with advanced experimentation if you like. Thanks for understanding.

Reviewer # 2 (Concern # 1)

It is suggested to cite the paper "Decision making Models on Perceived Uncertainty with Distributed Reinforcement Learning" .

Response: Respected Reviewer, we have cited this paper in the related work section with the following text lines:

In [1], the authors address the challenge of decision-making for autonomous vehicles in the presence of obstacle occlusions, proposing the Efficient-Fully parameterized Quantile Function (E-FQF) model. Using distributional reinforcement learning, the model optimizes for worst-case scenarios, improving decision efficiency and reducing collision rates compared to conventional reinforcement learning methods. Additionally, the model shows robustness in data loss scenarios, outperforming methods with embedded long and short-term memory.
Reviewer # 2 (Concern # 2)

As for the comment about "the algorithm proposed in this paper is not an optimal path in M2", this problem must be solved, in Fig. 10, the result is clearly not the optimal solution. You should at least obtain the optimal solution in the given environment, otherwise it cannot prove the correctness and effectiveness of the algorithm proposed in this paper.

Response: Respected Reviewer, there are many factors to determine the optimal path, number of turns is one of them. We have worked to get optimal path with respect to number of turns. The main purpose in this study is to find a better path than the traditional DQN approach [35] and it is evident form the results that the proposed However, if we run more episodes then the path received will eventually highly converged to be an ideal optimal path with minimum possible number of turns.

Reviewer # 2 (Concern # 3)

As for the comment "each environment only contains one obstacle map, and the map is static. It is unreasonable", designing a dynamic or stochastic environment may be too difficult. But in addition to "the steps we plan to take to address it", you should at least conduct experiments in some slightly more complex environments in this paper. You can refer to "An Online POMDP Solver for Uncertainty Planning in Dynamic Environment".

Response: We appreciate the reviewer's insightful observation. However, our study is conducted for the comparison with the base line research presented in [35] and that is a DQN algorithm, and the researchers in that published research (our baseline paper) has used the scope of static environment. We have adopted the both maps M1 and M2 from the base line research paper [35]. Moreover, the studies conducted in reinforcement learning area are always specific to a particular environment case and the environment case in the base line research paper [35] and in our study is an environment cluttered with static obstacles. Dynamic stochastic environment is not our scope in this study and neither the scope of our baseline paper. The respected reviewer's suggestion is already the part of our future plan for the said studies. We intend to continue this study for stochastic dynamic obstacles environment in the future.

Reviewer # 2 (Concern # 4)

As for the comment "The specific expression for the input state is still not clear", this article has not been modified in any way. This is necessary, please explain this in the paper.

Response: We appreciate the reviewer's insightful comments. The concern is corrected in the first paragraph of Section 2 Related Work and in the first paragraph of section Detailed Architecture of Deep Reinforcement Q-Learning. Moreover the missing information is also corrected in the Algorithm 1 Pseudo. The input of the deep Q-network is pixel positions, i.e., coordinates in the environment input image map and the output of the Q-network is a value function.

Reviewer # 2 (Concern # 5)

As for the comment about "fully connected dense layers", please also modify the "dense" in Fig. 3 to "Fully Connected".

Response: We are thankful for your feedback and appreciate your concern. Respected Reviewer, we have updated figure 3 in the paper.

Reviewer # 2 (Concern # 6)

As for the comment about "It is recommended to compare with algorithms such as DQN, TD3, SAC, etc", in addition to "the steps we plan to take to address it", you should at least use one method as baseline method in this paper.

Response: We appreciate the reviewer's insightful comment regarding the comparison. We have already compared it with DQN. DQN presented in reference cited at number [35] has been adopted as the baseline. A comparison with it is also provided in Table 5.

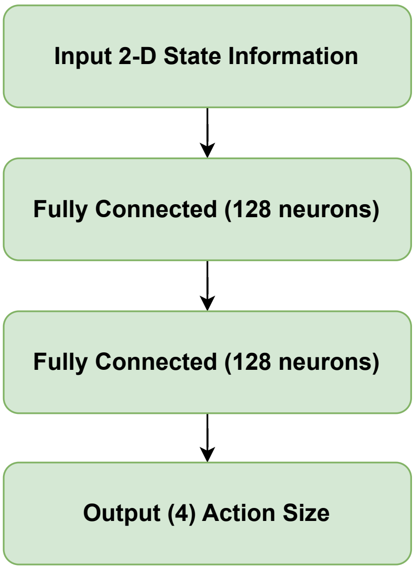

Figure 1: Network architecture.

References

[1] S. Xu, Q. Liu, Y. Hu, M. Xu, and J. Hao, "Decision-making models on perceptual uncertainty with distributional reinforcement learning," *Green Energy and Intelligent Transportation*, vol. 2, no. 2, p. 100062, 2023. [Online]. Available: https://www.sciencedirect.com/science/article/pii/S2773153722000627
